# Phytoconstituent analysis, anti-inflammatory, antimicrobial and anticancer effects of nano encapsulated *Convolvulus arvensis* L. extracts

**DOI:** 10.1186/s12906-024-04420-6

**Published:** 2024-03-14

**Authors:** Ezzat E. A. Osman, Mohamed A. Shemis, El-Sayed S. Abdel-Hameed, Abdullah E. Gouda, Hanem Hassan, Nahla Atef, Samah Mamdouh

**Affiliations:** 1 Department of Medicinal Chemistry, Theodor Bilharz Research Institute, Kornaish El-Nile St, Giza, 12411 Egypt; 2 Department of Biochemistry and Molecular Biology, Theodor Bilharz Research Institute, Kornaish El-Nile St, Giza, 12411 Egypt; 3 Air Force Specialized Hospital, Cairo, 19448 Egypt

**Keywords:** Alginate/chitosan nanoparticles, *C. arvensis*, Cytotoxicity, HepG2 cells, LC-ESI-MS analysis, Phenolic compounds

## Abstract

**Background:**

The *Convolvulus* genus is distributed all over the world and has a long history in traditional medicine. As nanotechnology expands its reach into areas like drug delivery and biomedicine, this study intends to assess the potential of *Convolvulus arvensis* L. extracts as anti-bacterial, anti-inflammatory and anti-cancer agents, along with chemical profiling of the methanolic (MeOH) extract active ingredients.

**Methods:**

The chemical composition of an 85% MeOH extract was investigated by liquid chromatography with an electrospray source connected to mass spectrometry (LC-ESI-MS). Both the 85% MeOH extract and *n*-butanol fraction of *C. arvensis* were loaded for the first time on alginate/chitosan nanoparticles. The 85% MeOH extract, *n*-butanol fraction and their loaded nanoparticles were tested for their cytotoxicity, anticancer, anti-inflammatory and antibacterial activity (against pathogenic bacteria, *E. coli* and *S. aureus*).

**Results:**

The chemical investigation of 85% MeOH extract of *C. arvensis* underwent LC-ESI-MS analysis, revealing twenty-six phenolic substances, of which 16 were phenolic acids, 6 were flavonoids, 1 glycolipid, 1 sesquiterpene and 2 unknown compounds. The FT-IR spectra confirmed the encapsulation of the 85% MeOH extract and *n*-butanol fraction onto alginate/chitosan nanoparticles and small size obtained by TEM maintained them nontoxic and enhanced their anti-inflammatory activity (the IC_50_ was decreased from 1050 to 175 µg/ml). The anti-cancer activity against HepG2 was increased and the cell viability was decreased from 28.59 ± 0.52 to 20.80 ± 0.27 at a maximum concentration of 1000 µg/ml. In addition, the MIC of encapsulated extracts was decreased from 31.25 to7.78 µg/ml in *E. coli* (Gm-ve) and from 15.56 to 7.78 µg/ml in *S. aureus* (Gm + ve) bacteria.

**Conclusion:**

Both alginate and chitosan are excellent natural polymers for the encapsulation process, which affects positively on the bioactive constituents of *C. arvensis* extracts and improves their biological properties.

## Introduction

Cancer remains a leading cause of mortality worldwide. Hepatocellular carcinoma (HCC), a prevalent form of cancer, predominantly emerges from cirrhosis due to chronic infections from hepatitis strains B, C, and D, excessive alcohol consumption, non-alcoholic steatohepatitis (NASH), and non-alcoholic fatty liver disease (NAFLD) [1, 2].

Treatment approaches for HCC have advanced over time. Given the multitude of treatment alternatives, collaboration among surgical, medical, and radiation oncology, hepatology, and interventional radiology is vital [3]. Deciding the optimal treatment is intricate due to frequently occurring liver cirrhosis and fibrosis, the disease’s scope, and HCC’s varying origins [4]. While curative treatments are scarce but crucial in cancer’s initial stages, many palliative treatments are available for middle-stage HCC. Lately, combined therapies have outperformed singular treatments in managing HCC, proving more effective in tumor control, enhancing survival rates, minimizing side effects, and helping overcome drug resistance [5].

In addition, antimicrobial agents are used for the treatment of infection dieseses; nevertheless, they have a number of negative side effects, such as elevating reactive oxygen species (ROS) that play a vital role in producing cancer [6]. Moreover,microbial infections lead to the inflammation, which is a natural response to physical injuries or exposure to harmful substances [7].

The appeal for plant-derived medicines, especially those rooted in traditional medicine, is rising in popularity [8]. Plants, especially those with ethnopharmacological and clinical uses have better patient tolerance and acceptance [9].

Recent advancements in nanoscience and nanotechnology have given rise to various synthetic metal nanoparticles (MNPs) [10, 11]. Currently, there’s an increasing trend of incorporating medicinal plants into nanoformulations to amplify their therapeutic effects [12].

Alginate is a natural polysaccharide extracted from brown algae and possesses many advantages compared to the various polymers adopted for elaborating hydrogel micro-and nanobeads. This natural, biodegradable and biocompatible material has a large variety of applications in functional formulations owing to its physical-chemistry properties, including gelling properties when cross-linked with di or trivalent ions such as calcium and aluminum cations such as Ca^+ 2^, Al^+ 3^, Ga^+ 3^ and La^+ 3^ [13, 14]. Both alginate and chitosan have been widely employed in the food, environmental and pharmaceutical industries for controlling medicine release [15].

The ionotropic gelation method can be used and the nanoparticles are produced by dropping a drug-loaded alginate solution into the aqueous solution of a soluble calcium salt, such as calcium chloride. The gelation of alginate is highly dependent on the concentration of CaCl_2_, due to the fact that Ca^+ 2^ ions are capable of binding to the carboxylic groups of alginate leading to the formation of a thermostable gel [16].

Chitosan is a biocompatible polysaccharide usually used in food industry, textiles and also medicine. Chitosan is made from chitin by deacetylation. Chitosan can achieve ionic interaction with sodium alginate. Calcium alginate nanoparticles were chosen because they are non-toxic and known for their hydrophilic carriers. Coating calcium alginate with chitosan can reduce the interaction between chitosan and extracts. This makes nanoparticles dissociate better [16–18].

*Convolvulus arvensis* L. (field bindweed) is a member of Convolvulaceae family [19]. This evergreen herbaceous weed, typically found in temperate, tropical, or Mediterranean regions, can spread as thick mats on the ground, with stems reaching up to 2 m. Historically recognized as both a food source and traditional medicine since the 18th century [20], *C. arvensis* has been widely used in folk medicine for ailments such as rheumatism, skin issues, infections, diabetes, and digestive disorders [21].

The previous studies reported that *C. arvensis* had several biological and pharmacological uses, including anti-tumor, antioxidant, anti-diabetic, hypertensive, anti-bacterial, anti-arthritic, hypoglycemic, anti-proliferative, and hepatoprotective activities [19, 22, 23]. In addition, the phytochemical analysis of the extracts of this plant demonstrated a large panel of bioactive phytoconstituents such as alkaloids, flavonoids, coumarins, phenolic acids, sterols and triterpenes [19, 24, 25]. The present study was aimed at evaluating the anti-inflammatory, anti-cancer, and anti-bacterial potential of *C. arvensis* nano-encapsulated and free extracts. In addition, investigate the chemical composition of the 85% MeOH extract using liquid chromatography integrated with an electrospray mass spectrometry method (LC-ESI-MS).

## Materials and methods

### Materials

Sigma-Aldrich (USA) provided the following substances: low viscosity sodium alginate, calcium chloride, chitosan (with a low molecular weight), deacetylated chitin (poly (D-glucosamine)), and glacial acetic acid. Loba Chemical (India) supplied phosphate buffer saline (PBS), sodium hydroxide, tween 80, and hydrochloric acid. All other chemicals used were of analytical grade. Ultrapure water with a resistivity of 18 µS/cm, obtained from a Millipore Milli-Q system in Milford, MA, USA, was used for all aqueous preparations.

### Collection and preparation of a plant sample

In March 2020, the aboveground portions of the *C. arvensis* plant were collected from Minia El-Qmah, El-Sharkia, Egypt. To ensure the plant’s identity, Professor Rim Hamdy, an expert in plant taxonomy and flora from the Department of Botany and Microbiology at Cairo University Herbarium, authenticated the plant sample. A voucher specimen labeled No. 25,320 was preserved at the Medicinal Chemistry Department of Theodor Bilharz Research Institute (TBRI). Subsequently, the plant materials were processed into a finely ground powder using a plant mill.

### Extraction and fractionation procedure

Five hundred grams of air-dried *C. arvensis* aerial parts were used to prepare an extract, via soaking the plant material in 85% MeOH in a conical flask. After this period, the mixture was filtered through Whatman filter paper No. 1 and then concentrated using a rotary evaporator (Buchi, Switzerland) under reduced pressure. The extraction process was repeated three times, resulting in a total of 65 g of methanolic extract.

Next, 50 g of the dried 85% MeOH extract were dissolved in water (100 ml) and subjected to sequential partitioning with petroleum ether (Pet-ether) (7 × 500 ml) and *n*-butanol (*n*-BuOH) (5 × 400 ml). Each solvent fraction was separately concentrated using the rotary evaporator until dryness was achieved. This process yielded a pet-ether fraction weighing 10.5 g, an *n*-BuOH fraction weighing 12.7 g, and a residue weighing 17.0 g. Each of these samples was stored in dry brown vials for future research purposes [26].

### Identification of the secondary metabolites via LC-ESI-MS

The study focused on analyzing the secondary metabolites present in an 85% MeOH extract of *C. arvensis* using LC-ESI-MS in negative ion mode. The LC system employed was a Waters Alliance 2695, featuring a C18 reversed-phase column with dimensions of 250 mm length and 5 μm particle size (Phenomenex, USA). The column was utilized with two eluents: water acidified with 0.1% formic acid (Eluent A) and a mixture of acetonitrile (CH_3_CN) and methanol (MeOH) in a 1:1 ratio, also acidified with 0.1% formic acid (Eluent B). A 20 µl injection of a 5 mg/ml sample was introduced, and elution occurred at a rate of 400 µl/min. The gradient program followed this sequence: 0.0–5.0 min (5% B), 5.0–10 min (5.0–10% B), 10–55 min (10–50% B), 55–65 min (50–95% B), and 65–70 min (5% B). The mass scan range was set from *m/z* 50 to 1000.

For mass spectrometry, specific conditions were applied: a cone voltage of 50 eV, a capillary voltage of 3 kV, a desolvation gas flow rate of 600 L/hr, a source temperature of 150 °C, a desolvation temperature of 350 °C, and a cone gas flow of 50 L/hr. Compound identification was accomplished based on retention time and comparison of mass spectroscopic data with existing literature data [26].

### Preparation of *C. arvensis* loaded alginate/CaCl_2_/chitosan nanoparticles

The process of preparing nanoparticles loaded with *C. arvensis* plant extracts, specifically the MeOH extract and *n*-BuOH fraction (NPs), was conducted following a modified method as described by Gandomi et al., 2016 [27]. To summarize, each plant extract (50 mg) was dissolved in 1 ml of phosphate buffer solution (PBS). The plant extract was added to 20 ml of a 0.3% alginate solution (ALG) and the mixture was mechanically stirred and pH adjusted to 5.3. After 30 min, 12 ml of a 0.2% calcium chloride solution were gradually introduced into the mixture. Following that, 8 ml of a 0.3% chitosan solution (CS) with a pH of 5.5 were added drop by drop, and the entire solution was subjected to magnetic stirring at 1000 rpm for an additional 30 min. Finally, the newly formed nanoparticles were separated by centrifugation at 13,000 rpm for 20 min. The same procedure was repeated without the inclusion of *C. arvensis* extracts to create blank nanoparticles, serving as a negative control. All nanoparticles were then frozen and subjected to lyophilization.

### Characterization of *C. arvensis* alginate/chitosan nanoparticles

#### Structural analysis of nanoparticles

The morphology and structural features of the prepared *C. arvensis* alginate/chitosan nanoparticles were analyzed using a transmission electron microscope (TEM) (Leo 0430, Leica, Cambridge, UK). TEM samples were prepared as follows, about 1 mg of each nanoparticle was dissolved in 1 mL distilled water, the sample was sonicated at 37 °C for 20 min to get a dispersed suspension, a small drop of the particle suspension was placed onto a carbon-coated copper grid and dried completely. Then, the grid was tapped with filter paper to remove any excess water. Finally, images were taken using a transmission electron microscope.

The characterization of *C. arvensis* alginate/chitosan nanoparticles also involved assessing their size distribution, zeta potential, and polydispersity index (PDI). This analysis was conducted using a Malvern Zetasizer Nano ZS instrument (Malvern Instruments Ltd., Malvern, Worcestershire, UK). In this process, 2 mg of each nanoparticle (NP) was resuspended in a 2 ml solution of 1% acetic acid. To ensure a consistent dispersion, the solution was subjected to sonication for 10 min and then filtered through a membrane syringe filter with a pore size of 0.22 mm. The determination of particle size was performed in triplicate at a temperature of 25 °C, using an angle of 90° for the photomultiplier, and a wavelength of 633 nm. To ascertain the surface charge, known as zeta potential, electrophoretic mobility measurements were taken. These zeta potential measurements were conducted in triplicate at pH 5.5 using a 100 𝜇l aqueous dip cell with the Zetasizer Nano ZS instrument (Malvern Instruments Ltd., Malvern, Worcestershire, UK). Prior to measurement, the samples were diluted 1:100 with double-distilled water.

#### FTIR analysis

The Fourier transform infrared (FTIR) spectra of MeOH, *n-*BuOH, unloaded Alg/Cs NPs, MeOH-NPs and *n-*BuOH-NPs were obtained using Bruker VERTEX 80 (Germany), supplied with a diamond disk as that of an internal reflector in the range 4000 − 400 cm^− 1^ with resolution 4 cm^− 1^, and reflective index 2.4.

#### TGA analysis

The thermogravimetric analysis (TGA) curves of all nanoparticles were recorded using a TGA thermogravimetric analyzer (micro) (TGA-50/50H, Shimadzu, Columbia, USA) thermo-balanced in a temperature range of 10–802 °C at a heating rate of 10 K/min under nitrogen.

### Anti-inflammatory activity using protein denaturation assay

This assay operates on the premise that when albumin protein denatures, it produces antigens that trigger a type III hypersensitive reaction, resulting in inflammation. A variety of extracts were studied in vitro for their effects on protein denaturation using either egg or bovine serum albumin (BSA) [28, 29].

5 ml solution containing 2.8 ml of freshly prepared PBS (pH 6.3), 2 ml of a specific concentration of *C. arvensis*, *C. arvensis*-alginate/chitosan-NPs extracts, alginate/chitosan-NPs and 0.2 ml of BSA. A serial dilution of *C. arvensis*, *C. arvensis*-alginate/chitosan-NPs extracts and alginate/chitosan-NPs were prepared as follows: 1000, 500, 250, 62.5, 31.25 and 15.56 µg/ml. Diclofenac sodium served as the reference compound and was tested at the same concentrations, ranging from 1000 to 15.56 µg/ml. The various mixtures were first warmed in a water bath at 37ºC for 20 min. The temperature was then incrementally raised to 70ºC in 5-minute intervals. Once the heating was complete, the samples were allowed to cool to room temperature. Their optical density was subsequently measured at 660 nm wavelength. Each experiment was repeated three times to ensure accuracy. The degree to which protein denaturation was inhibited was computed using a specific formula.

Inhibition %= (Abs _Control_ – Abs _Sample_) × 100/ Abs _control_.

### The cytotoxic activity using membrane stabilization and red blood cells toxicity assays

When the drug administrated (herbal extract) and circulates into the blood stream, red blood cells (RBCs) appear to be the first biological moieties being affected. Therefore, the RBCs toxicity (hemolysis) assay can be used to determine the degree of toxicity of the *C. arvensis, C. arvensis*-alginate/chitosan-NPs extracts and alginate/chitosan-NPs on the RBCs.

### Preparation of red blood cells suspension

Blood samples were taken from healthy participants who hadn’t consumed any anti-inflammatory medications for two weeks. After centrifugation at 3000 rpm for 10 min, the plasma was removed. The RBCs were then rinsed three times with an equivalent amount of saline. The volume of the blood was measured and reconstituted as 10% v/v suspension with normal saline. Different concentrations of *C. arvensis*, *C. arvensis*-alginate/chitosan-NPs extracts and alginate/chitosan-NPs were prepared as follow; 1000, 500, 250, 125, 62.5, 31.25 and 15.56 µg/ml. To each 400 µl sample, 100 µl of the erythrocyte suspension (derived from 1 ml of densely packed cells mixed in 10 ml PBS) was added. For controls, a PBS solution and a 10% v/v solution of Triton X-100 were used as negative and positive controls, respectively. After an incubation period of 4 h at 37 °C, the samples underwent centrifugation for 10 min at 2000 rpm. The supernatant was then collected, and the extent of hemolysis was inferred from the optical density of the released hemoglobin at 540 nm using a UV-VIS spectrophotometer (Abbott, Kinetic Spectrophotometer, New Jersey, USA). Hemolysis percentages were then calculated based on a standard equation, considering the hemolysis in the control as 100%.

Hemolysis %= (Abs_sample_ –Abs_neg_ /Abs_pos_) × 100%.

The terms in this equation represent the following: Abs_sample_ is the optical reading of the sample; Abs_neg_ represents the optical reading of the negative control; and Abs_pos_ signifies the optical reading of the positive control.

### Cytotoxicity assay

#### Cell lines and culture conditions

All Vero cell lines (ATCC-C1008 obtained from the Department of Cell Culture, Vacsera, Egypt) were grown in MEM media supplemented with 10% heat inactivated (FBS and 100 IU/ml penicillin and 100 IU /ml streptomycin). Cultures were maintained in a humidified atmosphere with 5% CO_2_ at 37 °C. The cells were dispensed into 96-well culture plates at a density of 5 × 10^4^ cells/ml and left for 24 h at 37 ºC to achieve approximately 70% confluence. Before the analysis of the *C. arvensis, C. arvensis*-alginate/chitosan-NPs extract and alginate/chitosan-NPs, cells were rinsed with PBS then replenished with a fresh medium. Cells were treated with15.62-1000 µg/ml of each test substance. The stock solutions of the tested samples were prepared using absolute PBS and diluted using culture media. After 24 h, the medium containing the drug control (Doxorubicin) and plant extract was removed and the cells were washed with PBS, then 50 µl of 0.5% crystal violet staining solution were added to each well, and incubated for 20 min at room temperature on a bench rocker with a frequency of 20 oscillations/min.

The plates were washed four times in a stream of tap water and inverted on filter paper to remove any remaining liquid. The plates were air-dried for at least 2 h at room temperature. Then, 200 µl of methanol were added to each well and incubated for 20 min at room temperature on a bench rocker with a frequency of 20 oscillations/min. The optical density of each well was measured at 570 nm (OD_570_) with a plate reader.

Cell viability %= (Abs_sample_ - Abs_blank_/ Abs_mc_ - Abs_blank_) × 100.

Where Abs_sample_ is the absorbance of the sample, Abs_blank_ is the absorbance of the blank and Abs_mc_ is the absorbance of the control medium.

### Anti-cancer assay

#### Cell lines and culture conditions

Similar to the Vero cell lines, the HepG2 cell lines (ATCC-CCL 75 obtained from the Department of Cell Culture, Vacsera, Egypt) were cultivated in RPMI-1640 media which was enriched with 10% heat-inactivated fetal bovine serum (FBS) and the same concentrations of antibiotics. These cells were also cultured in a humidified setting with 5% CO_2_ at 37 °C. The cells were then plated in 96-well culture plates at the same density and incubated for 24 h at 37 °C until around 70% confluence was reached. Before the analysis of the *C. arvensis*, *C. arvensis*- alginate/chitosan-NPs *extract* and alginate/chitosan-NPs, cells were washed with PBS and given fresh media. Cells were treated with 15.62–1000 µg/ml of each test substance. The tested sample stock solutions were prepared using PBS and diluted using culture media. After a 24-hour period, the cells, which had been exposed to Doxorubicin and other samples, were rinsed with PBS. Next, each well received 50 µl of a 0.5% crystal violet solution, and the mixture was left to sit for 20 min at room temperature, being gently rocked at 20 shakes per minute. Following this, the plates were rinsed four times with tap water and left upside down to drain on filter paper. Once they had dried in the open air for roughly 2 h, each well was filled with 200 µl of methanol. This setup was again left for 20 min at room temperature, undergoing the same gentle rocking motion. Finally, the optical density of the wells was gauged at 570 nm using a plate reader.

Cell viability %= (Abs_sample_ - Abs_blank_/ Abs_mc_ - Abs_blank_) ×100.

In this formula, Abs_sample_ denotes the optical reading of the sample, Abs_blank_ stands for the optical reading of the blank, and Abs_mc_ represents the optical reading of the control medium.

#### Anti-bacterial effects of *C. arvensis* extracts

The *C. arvensis*, *C. arvensis*-alginate/chitosan-NPs extracts and alginate/chitosan-NPs were subjected to anti-bacterial evaluation against two bacterial strains *E. coli* (ATCC 8739) and *S. aureus* (ATCC 25,923). The extracts of *C. arvensis* and the prepared NPs were tested for the minimum inhibitory concentration (MIC) by the well diffusion method. The samples were diluted to various concentrations (15.62–250 µg/ml) using PBS. The bacteria, grown for 24 h in nutrient agar, were evenly spread over the medium using the spread plate technique, at a density of 10 µl (106 cells/ml). Once the medium solidified, wells of 5 mm diameter were filled with the nanoparticle-infused extracts. For comparison, Ceftriaxone, at a concentration of 10 µg/ml, was used as a standard control. After incubating these plates at 37 °C for a day, the extent of bacterial growth inhibition was assessed by measuring the diameters of the clear zones around the wells.

## Results

### LC-ESI-MS secondary metabolites analysis of *C. arvensis* 85% MeOH extract

**Fig. 1:**
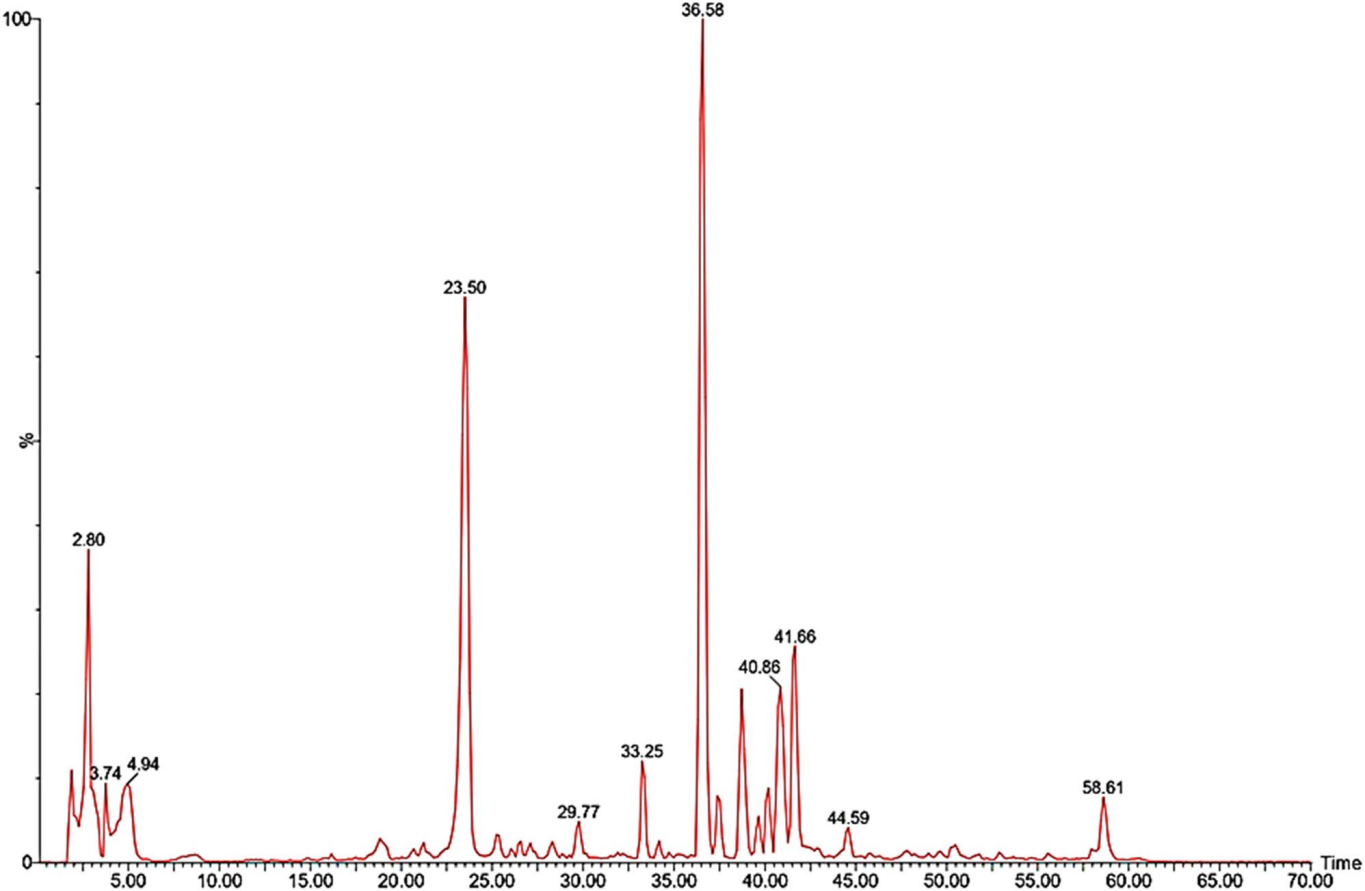
LC-ESI-MS total ion chromatogram (TIC) of the 85% MeOH extract of *C. arvensis*

**Fig. 2:**
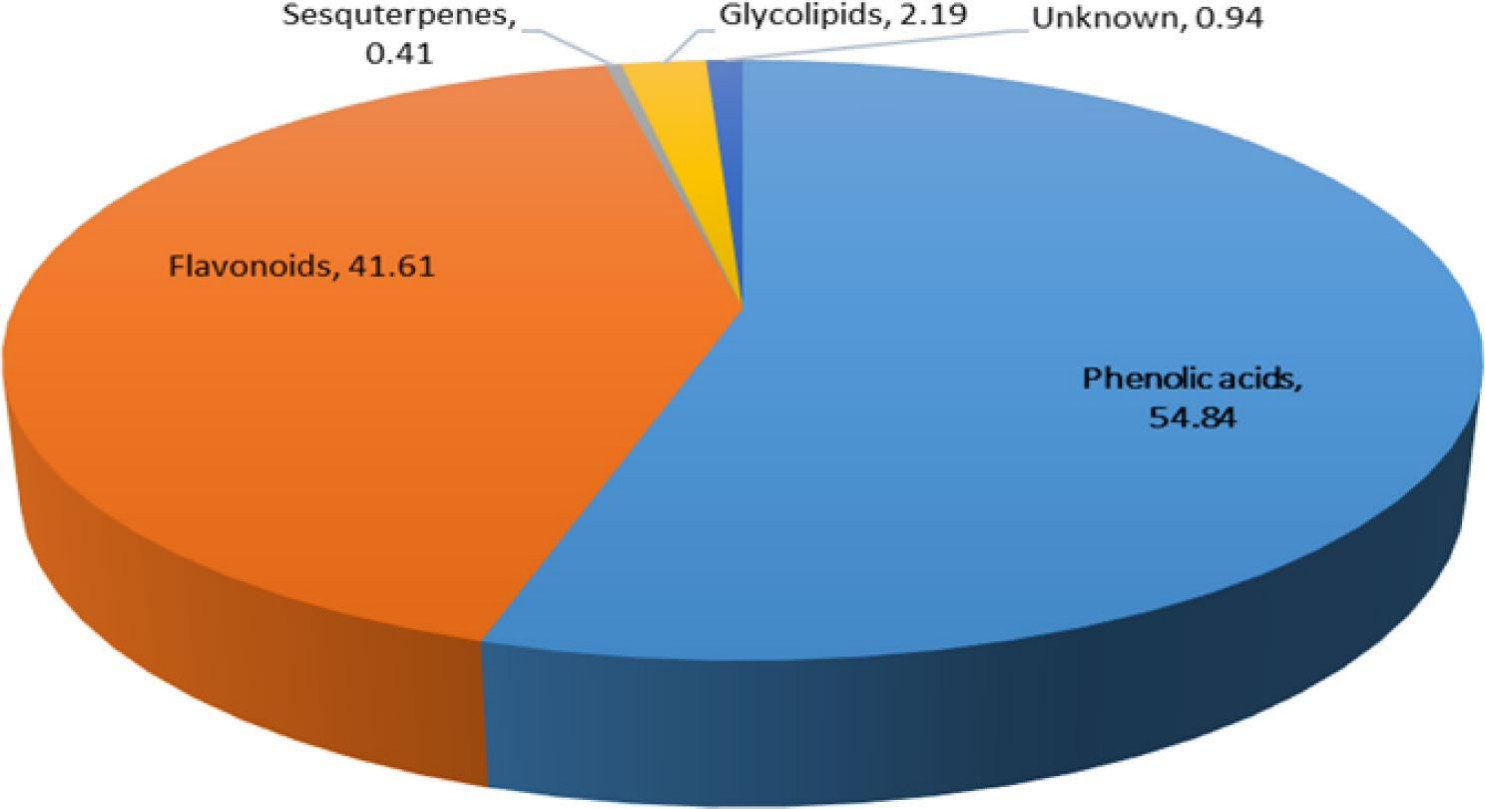
Pie chart of the major chemical classes tentatively identified in *C. arvensis* 85% MeOH extract in terms of area (% of the total area) in the TIC in negative ion mode

**Fig. 3:**
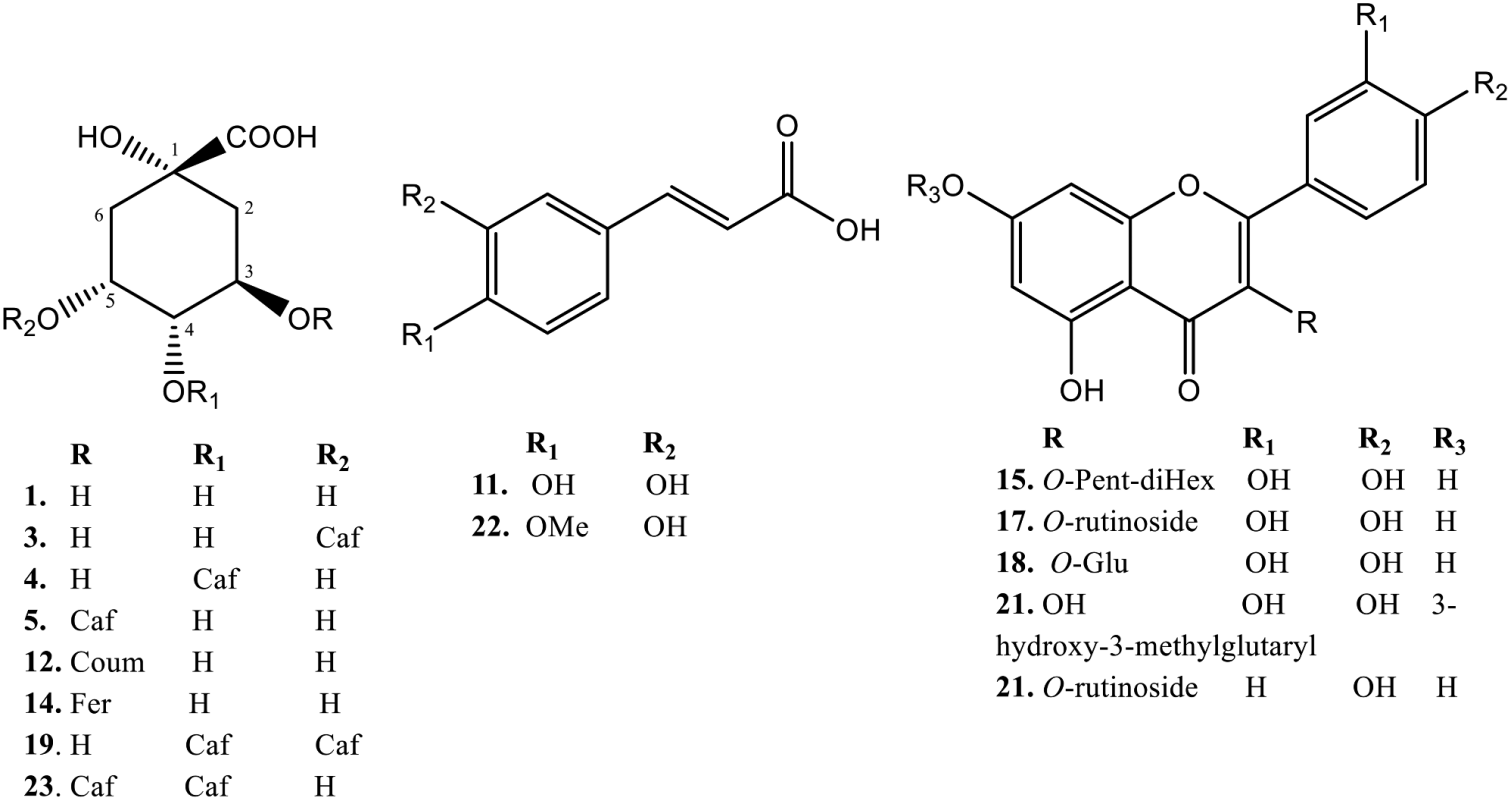
The chemical structures of some assigned compounds

The secondary metabolite analysis of the 85% MeOH extract of *C. arvensis* was carried out by LC-ESI-MS. The plant secondary metabolites were characterized by comparing their data with the literature. A total of 26 polyphenolic compounds were categorized as 16 phenolic acids, 6 flavonoids, 1 glycolipid, 1 sesquiterpene and 2 unknown compounds (Table 1). Figure 1 shows the total ion chromatograms of 85% MeOH extract, the peaks inside the chromatogram refer to the identified compounds as mentioned in Table 1 and the numbers over these peaks correspond to the retention time (t_R_). In addition, Fig. 2 represents the Pie chart of the major chemical classes of identified compounds in terms of area under the peak in the following order; phenolic acids (54.84%) followed by flavonoids (41.61%), glycolipids (2.19%) unknown compounds (0.94%), and sesquiterpenes (0.41%). Moreover, Fig. 3 shows the chemical structures of some assigned compounds.

**Table 1:** Tentative assignment of chemical constituents of 85% MeOH extract of *C. arvensis* by LC-ESI-MS

Identified compound	t_R_ (min)	M. Wt	[M-H]^−^	*m/z* fragments	Area %
**Phenolic acids**					
Quinic acid	12.02	192	191	173, 97	0.02
Benzoyl-Caffeoylquinic acid	16.16	476	475	353, 191, 135	0.11
5-*O*-Caffeoylquinic acid	18.83	354	353	191 (100%), 179 (63%), 135	0.97
4-*O*-Caffeoylquinic acid	19.10	354	353	191 (40%), 179 (66%), 135	0.03
3-*O*-Caffeoylquinic acid dimer	20.70	708	707	353, 191 (100%), 135	0.15
5-*O*-Caffeoylquinic acid dehydrodimer	21.23	706	705	513, 339, 191	0.27
5-*O*-Caffeoylquinic acid dimer	23.50	708	707	353, 191 (100%), 179, 135	29.42
5-*O*-Caffeoylquinic acid dimer isomer	25.23	708	707	353, 191 (100%), 179, 135	0.79
4-*O*-Caffeoylquinic acid dimer	26.04	708	707	191(40%), 179, 135 (100%)	0.19
Caffeic acid	27.10	180	179	161, 135	0.23
Coumaroylquinic acid	28.30	338	337	191, 173, 93, 85	0.41
Feruluylquinic acid	29.77	368	367	191, 173	1.24
4,5-di-*O*-caffeoylquinic acid	38.72	516	515	353, 191, 179, 135	5.81
Ferulic acid	40.86	194	193	161, 133	7.74
3, 4-di-*O*-caffeoylquinic acid	41.67	516	515	353, 191, 179, 135	6.91
Caffeic acid derivatives	50.47	424	423	285, 179, 161	0.55
**Flavonoids**					
Quercetin-*O-*pentosyl-dihexosides	33.25	742	741	609, 301, 179	2.64
Apigenin-C-hexoside-*O*-pentoside	34.18	564	563	443, 413, 293	0.31
Quercetin-3-*O*-rutionside (Rutin)	36.58	610	609	463, 301, 179, 151	33.63
Quercetin-3-*O*-hexoside	37.39	464	463	301, 179, 151	1.73
Quercetin-7-*O*-[3-hydroxy-3-methylglutaroyl] hexoside	39.65	308	607	505, 463, 301	1.10
Kaempferol-3-*O*-rutinoside	40.19	594	593	285	2.20
**Glycolipids**					
Trihydroxy-10,15-octadecadienoic acid derivative	58.61	328	327	291, 229, 211, 171	2.19
**Sesquiterpenes**					
Magastigmane glycoside derivative	26.57	388	387	161	0.41
**Unknown compounds**					
Unknown	28.84	622	621	511, 337, 191	0.09
Unknown	44.59	432	431	313, 191, 179, 149 (100%), 135	0.85
**Total area % of identified compounds**					**99.99**

### Characterization of *C. arvensis* alginate/chitosan nanoparticles

**Fig. 4:**
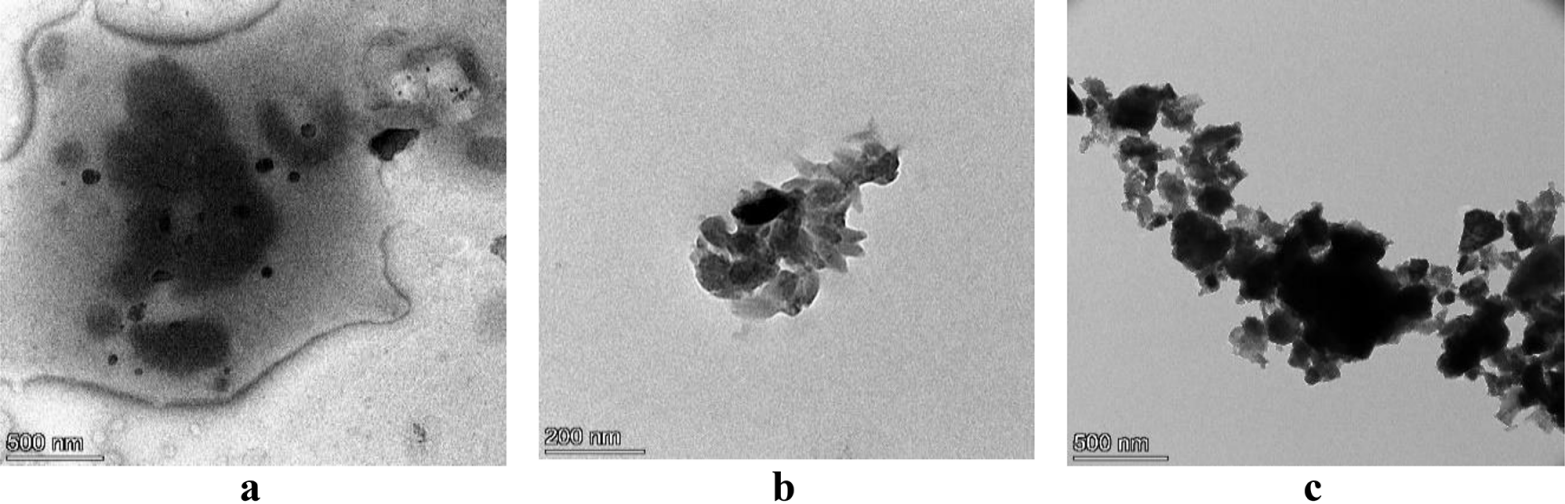
The particle size of the unloaded Alg/Cs-NPs (**a**), MeOH-NPs (**b**), *n*-BuOH -NPs (**c**)

The results of the transmission electron microscopy confirmed that the nanometric size and the spherical morphology of the prepared unloaded Alg/Cs-NPs were (55 ± 3.9 nm) and the subspherical (cubic) morphology of MeOH-NPs (90 ± 4.8 nm) and *n-*BuOH-NPs (92 ± 5.5 nm) as shown in Fig. 4a and b, and 4c. TEM analysis showed the capsular structure of the NPs due to the novel method, as it shows that the NPs take a spherical shape (chitosan coat) with an inner dark portion of the drug encapsulated (calcium alginate) in a polymer shell, which is bounded by the outer smooth layer of stabilizer that keeps the particles from aggregation and all NPs have an amorphous structure. Whereas the spherical morphology was changed into a subspherical or cubic structure after loading both the MeOH extract and *n*-BuOH fraction.

**Fig. 5:**
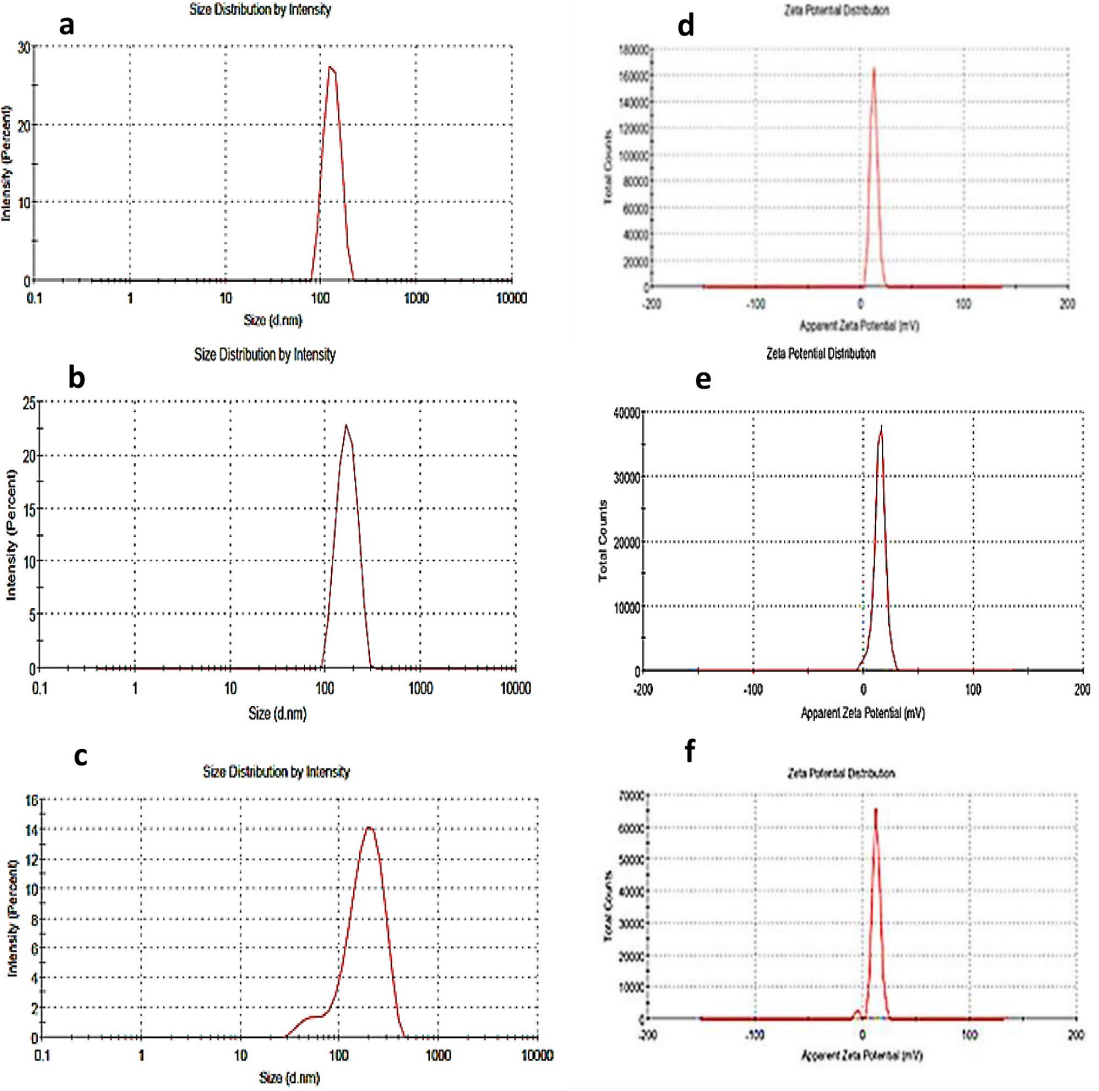
The particle size of the free Alg/Cs-NPs (**a**), *n*-BuOH-NPs (**b**), MeOH-NPs (**c**), and the zeta potential of the particles (**d**, **e** and **f**)

The dynamic light scattering illustrated the homogenous particle size distribution of free polymers and *C. arvensis*-polymers NPs, where the DLS particle size patterns showed one peak at 132.2, 171.5 and 188.7 nm for the free Alg/Cs-NPs, *n*-BuOH-NPs and MeOH-NPs with zeta potential of 13.3, 14.9 and 12.6 mV, respectively as represented in Fig. 5.

### FTIR Analysis

**Fig. 6:**
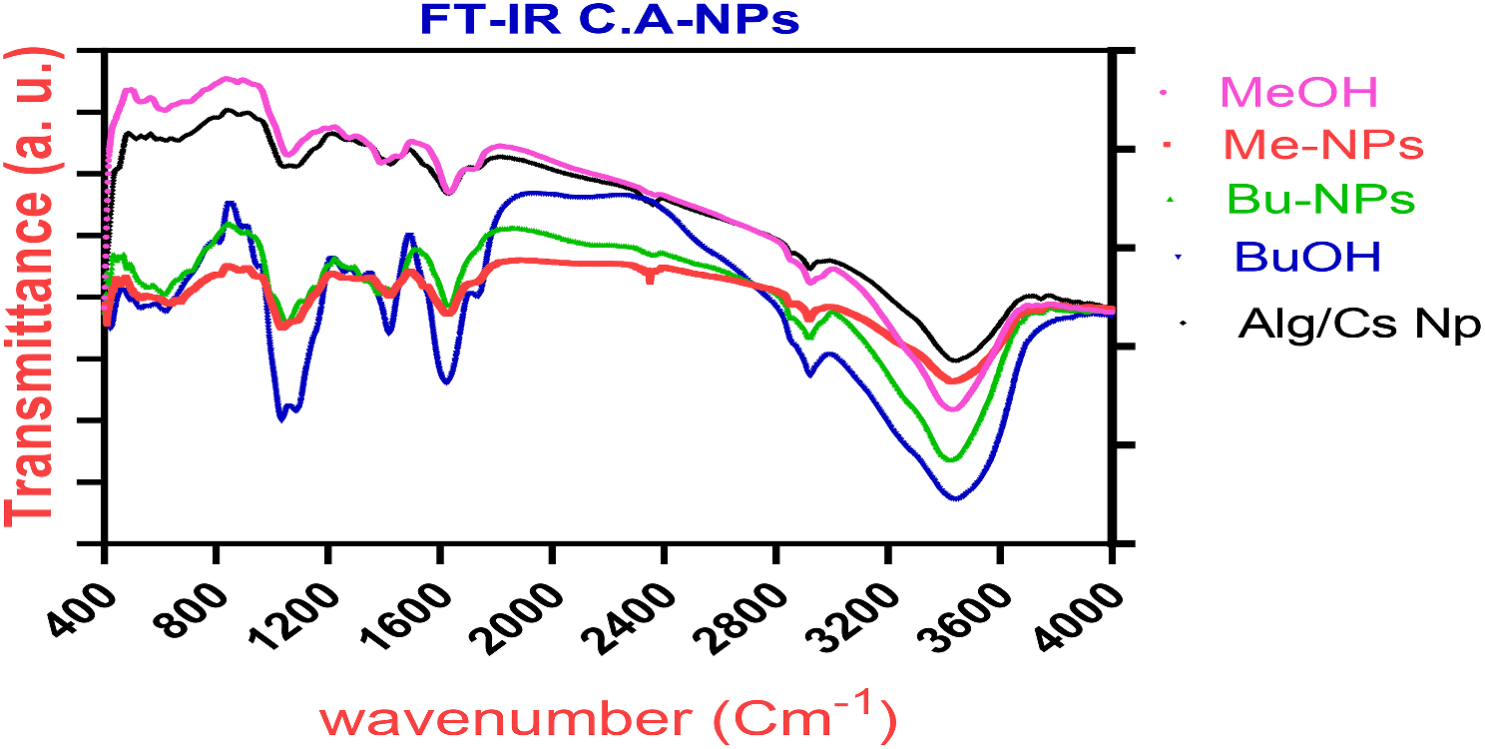
FTIR spectra of (**a**) Alg/Cs-NPs (**b**) *n*-BuOH (**c**) MeOH (**d**) MeOH-NPs (**e**) *n*-BuOH-NPs

The FTIR spectra of unloaded Alg/Cs-NPs, MeOH-NPs and n-BuOH-NPs were as follows: unloaded Alg/Cs-NPs IR spectra revealed characteristic absorption bands at wavelengths of 3746 cm^− 1^ (NH2 and -OH groups stretching), 1650 cm^− 1^ (amide I), 1470 cm^− 1^ (Amide II), and 1357 cm^− 1^ (Amide III). The characteristic peaks that appeared at 3446 cm^− 1^ represent the -OH group, the stretching vibrations at 1031 cm^− 1^ indicate the presence of C-O stretch of cyclic alcohols, and the stretching vibrations at 1089.5 cm^− 1^ correspond to asymmetric and symmetric -COO- stretching vibration of carboxylate groups. The sodium alginate’s saccharide structure is thought to be responsible for the strong band at 920 cm ^− 1^ (C-O-C stretching). The vibration bands at 3429.7 cm^− 1^ suggest -OH stretching in non-encapsulated MeOH extract. and *n*-BuOH. The peaks at 2915.84 cm^− 1^ (aliphatic C-H stretching), 1621 cm^−1^, and 1418 cm^− 1^ (aromatic C = C stretching) in both MeOH extract. and *n*-BuOH. Alg/Cs polymeric network enclosed the MeOH ext. and *n*-BuOH, resulting in the powerful peak at 2349 cm^− 1^ and 935 cm^− 1^. Also, intense peaks at 1615 cm-1 and 1725 cm^− 1^ in the spectra of the MeOH ext. and *n*-BuOH were diminished in final build of encapsulated nanoparticles because of being physically confined in the polyelectrolyte complex, as demonstrated by FTIR, without forming any chemical complexes with the polymers as shown in Fig. 6.

### TGA analysis

**Fig. 7:**
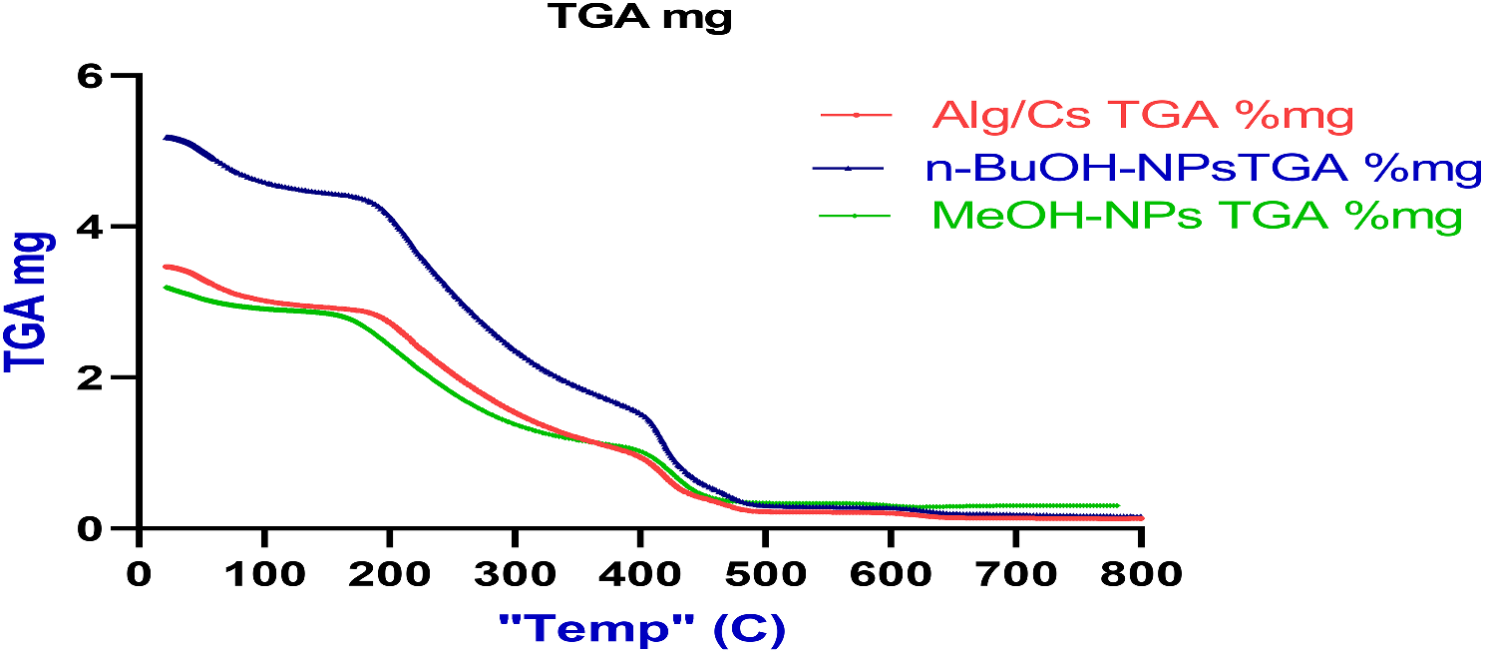
TGA curves of Alg/Cs-NPs, MeOH-NPs and *n*-BuOH-NPs

The thermostability of the Alg/Cs, nanoparticles without *C. arvensis* and nanoparticles loaded *C. arvensis* MeOH and *n*-BuOH extracts, was evaluated using thermogravimetric analysis (TGA) as shown in Fig. 7. Compared with the unloaded Alg/Cs nanoparticles, a lower thermal degradation temperature for both MeOH and *n*-BuOH-NPs. The MeOH-NPs had the lowest weight loss followed by *n*-BuOH-NPs and finally unloaded Alg/Cs-NPs, -9.85%, -13.83 and − 15.55% at temperature (22.65- 139.82^◦^C) and − 90.09%, − 97.01% and − 96.18 at temperature (512.34-800.11^◦^C) for the three nanoparticles respectively. The weight loss at the medium temperatures was − 48% (139.82-339.67^◦^C) and − 31.6% (339.67-512.34^◦^C) for all nanoparticles.

### Anti-inflammatory activity of *C. arvensis* extracts

**Fig. 8:**
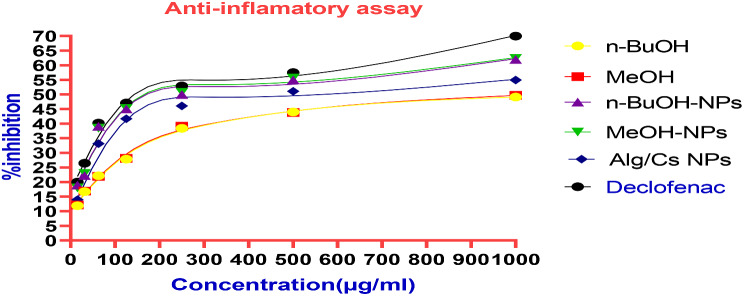
Anti-inflammatory activity of free Alg/Cs-NPs, *n-*BuOH-NPs and MeOH-NPs and standard (Diclofenac sodium)

Protein denaturation can lead to various inflammatory diseases, including diabetes, cancer, and rheumatoid arthritis. Reducing inflammation can be achieved by preventing this protein denaturation. The current research highlighted the ability of *C. arvensis, C. arvensis*-alginate/chitosan-NPs extracts, and alginate/chitosan-NPs to combat inflammation in vitro by obstructing protein denaturation. They effectively halted heat-induced albumin denaturation in a manner dependent on concentration, suggesting their capability to manage protein denaturation associated with inflammation (Table 2; Fig. 8). The IC_50_ of *C. arvensis* (MeOH ext. and *n*-BuOH fr.), *C. arvensis*-alginate/chitosan-NPs extracts, free alginate/chitosan-NPs and standard (Diclofenac sodium) were found to be 1050, 175, 555 and 143 µg/ml respectively. The results of this study indicate that the encapsulation of *C. arvensis* extracts onto alginate/chitosan-NPs enhances their anti-inflammatory activity.

**Table 2:** Anti-inflammatory activity of *C. arvensis*, *C. arvensis*-alginate/chitosan-NPs, alginate/chitosan-NPs and standard (Diclofenac sodium)

Concentration (µg/ml)	% Inhibition					
	*n*-BuOH	MeOH	*n*-BuOH- NPs	MeOH- NPs	Alg/Cs Np	Diclofenac sodium
1000	49.11 ± 1.09	49.71 ± 0.88	61.98 ± 2.02	62.38 ± 2.14	54.93 ± 0.74	69.96 ± 1.46
500	44.01 ± 1.41	43.81 ± 1.71	54.98 ± 1.59	55.61 ± 1.73	51.07 ± 0.89	57.49 ± 1.02
250	38.42 ± 0.77	39.12 ± 0.84	49.95 ± 0.89	50.75 ± 1.09	46.08 ± 1.22	52.89 ± 1.51
125	27.83 ± 1.05	28.13 ± 1.15	44.99 ± 0.84	45.19 ± 0.65	41.69 ± 0.57	47.06 ± 0.69
62.5	22.16 ± 0.78	21.96 ± 0.73	39.06 ± 0.68	38.46 ± 0.61	33.16 ± 0.49	40.17 ± 1.18
31.25	16.69 ± 0.71	16.92 ± 0.78	22.19 ± 1.29	22.99 ± 1.11	16.85 ± 0.42	26.42 ± 0.51
15.56	11.88 ± 0.79	12.18 ± 0.74	18.94 ± 0.480	17.99 ± 0.42	13.97 ± 0.41	19.97 ± 0.78
IC_50_ (µg/ml)	1050	1050	175	175	555	143

### Cytotoxic activity of *C. arvensis* extracts

#### Membrane stabilization assay and RBCs toxicity

**Fig. 9:**
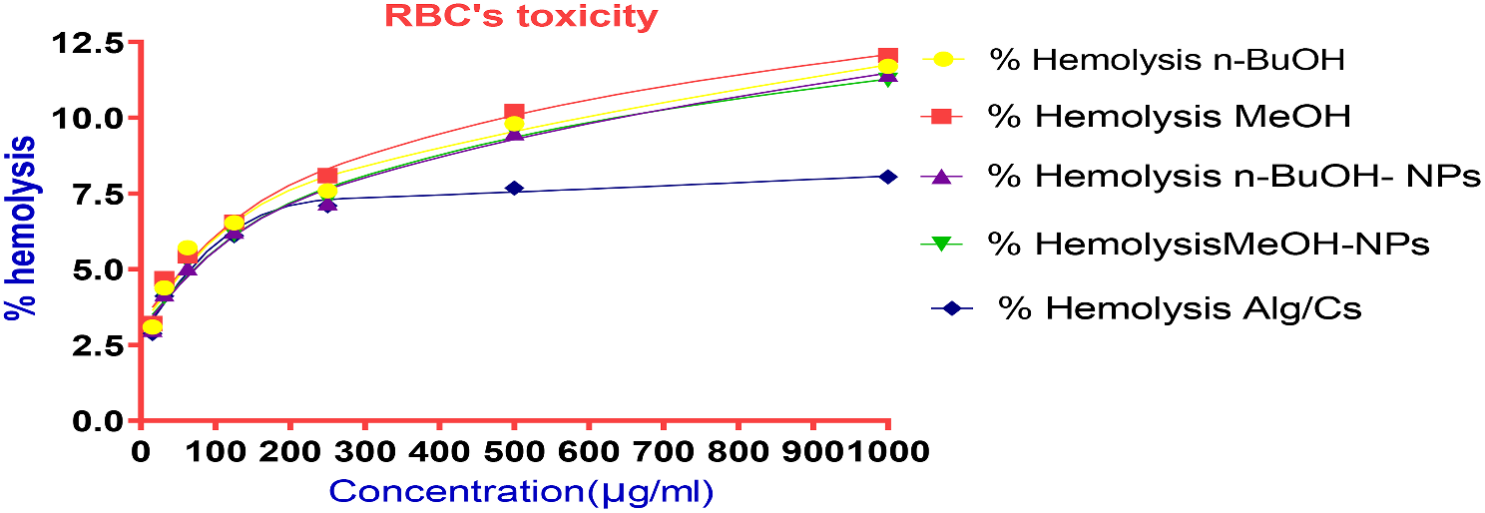
Hemolysis %of RBC’s treated with free Alg/Cs-NPs, *n*-BuOH-NPs and MeOH-NPs

All the *C. arvensis*, *C. arvensis*-alginate/chitosan- NPs, alginate/chitosan- NPs showed a slight effect on membrane stabilization (Table 3; Fig. 9). It was observed that the encapsulation of *C. arvensis* extracts onto alginate/chitosan-NPs did not increase the hemolysis of red blood cells. The percent of hemolysis was maintained around 3–12% even at maximum concentration of 1000 µg/ ml for each tested parameter.

**Table 3:** The hemolytic effect of *C. arvensis*, *C. arvensis*-alginate/chitosan-NPs, alginate/chitosan-NPs on the RBC’s

Concentration (µg/ml)	% Hemolysis				
	*n*-BuOH	MeOH	*n*-BuOH- NPs	MeOH- NPs	Alg/Cs Np
1000	11.7	12.06	11.44	11.24	8.05
500	9.8	10.21	9.51	9.56	7.68
250	7.59	8.09	7.19	7.29	7.09
125	6.53	6.55	6.25	6.15	6.11
62.5	5.709	5.44	5.04	5.24	5.44
31.25	4.388	4.69	4.19	4.09	4.11
15.56	3.09	3.21	3.02	3.12	2.88
IC_50_ (µg/ml)	-------	-------	-------	-------	-------

### Cytotoxicity assay on Vero cell line

**Fig. 10:**
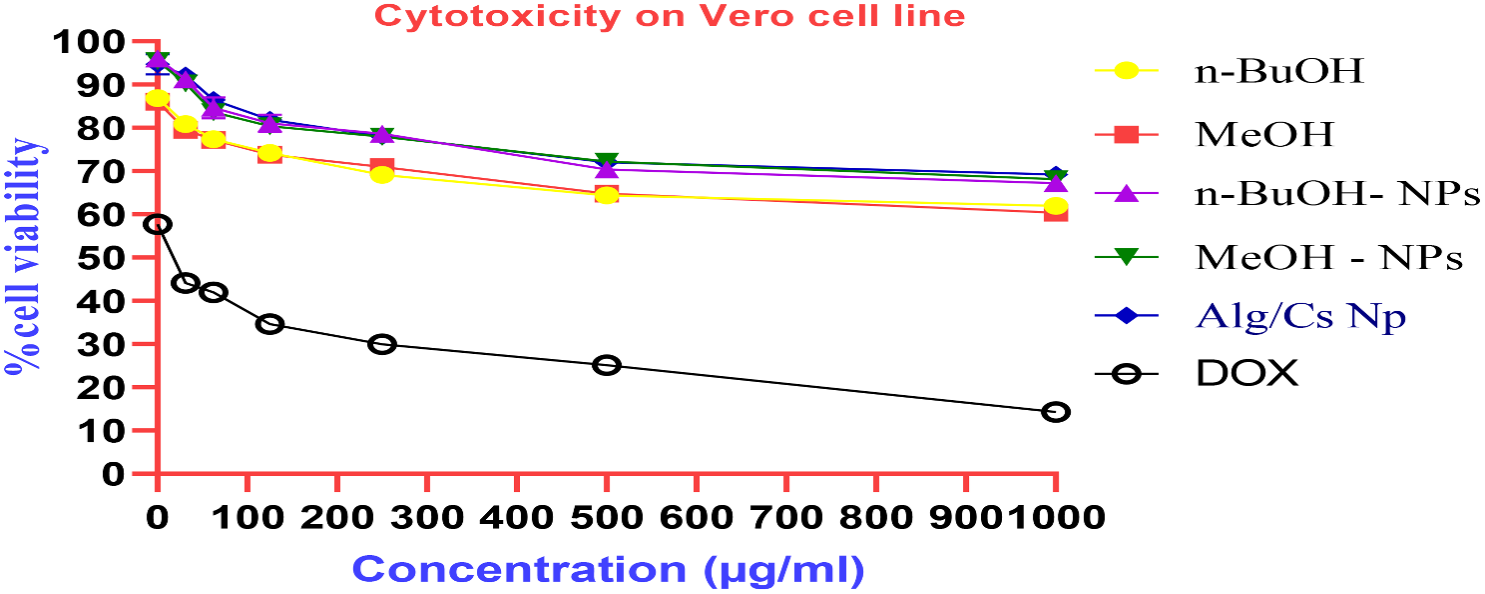
Viability % of Vero cells treated with free Alg/Cs-NPs, *n*-BuOH-NPs and MeOH-NPs and standard (Doxorubicin)

The cytotoxicity of *C. arvensis*, *C. arvensis*-alginate/chitosan-NPs, alginate/chitosan-NPs, and the standard (Doxorubicin) was assessed in vitro on Vero cell lines after 24 h. IC_50_ values were derived from cell viability graphs over concentrations ranging from 15.62 to 1000 µg ml^− 1^. A line chart was constructed using GraphPad Prism 8, mapping concentrations (X-axis) against % cell viability (Y-axis). The concentration corresponding to 50% inhibition on the Y-axis was then identified (Table 4; Fig. 10).

**Table 4:** The cytotoxic effect of *C. arvensis*, *C. arvensis*-alginate/chitosan-NPs, alginate/chitosan- NPs and standard (Doxorubicin) on the Vero cell lines

Concentration (µg/ml)	% cell viability					
	*n*-BuOH	MeOH	*n*-BuOH- NPs	MeOH- NPs	Alg/Cs Nps	DOX
1000	61.97 ± 1.40	60.40 ± 1.23	67.23 ± 1.23	68.09 ± 1.58	69.23 ± 1.41	14.25 ± 0.62
500	64.39 ± 1. 23	64.82 ± 0.88	70.37 ± 1.92	72.22 ± 1.26	72.08 ± 1.65	25.09 ± 0.57
250	69.09 ± 1.92	70.94 ± 1.75	78.63 ± 1.74	77.94 ± 1.95	78.07 ± 1.22	29.99 ± 0.41
125	74.22 ± 1.56	73.79 ± 1.41	81.05 ± 1.98	80.34 ± 1. 39	81.91 ± 1.06	34.59 ± 0.79
62.5	77.35 ± 1.40	77.21 ± 1.01	84.62 ± 1.45	83.62 ± 1. 76	86.47 ± 1.57	41.96 ± 0.87
31.25	80.91 ± 0.88	79.50 ± 1.59	91.17 ± 1.41	90.31 ± 1.41	92.16 ± 1.75	44.09 ± 1.55
15.56	86.89 ± 1.41	86.04 ± 1.41	96.01 ± 1.23	95.44 ± 1.41	94.73 ± 2.32	57.73 ± 0.94
IC_50_ (µg/ml)						22.09

The results revealed that *C. arvensis*, *C. arvensis*-alginate/chitosan- NPs, alginate/chitosan- NPs exhibited non-cytotoxic activities varying compared to the common anti-cancer drug, Doxorubicin. It was also noticed that the encapsulation of *C. arvensis* extracts onto alginate/chitosan-NPs increased the viability of Vero cell lines (Table 4; Fig. 10).

### Anti-cancer assay on HepG_2_ cell line

**Fig. 11:**
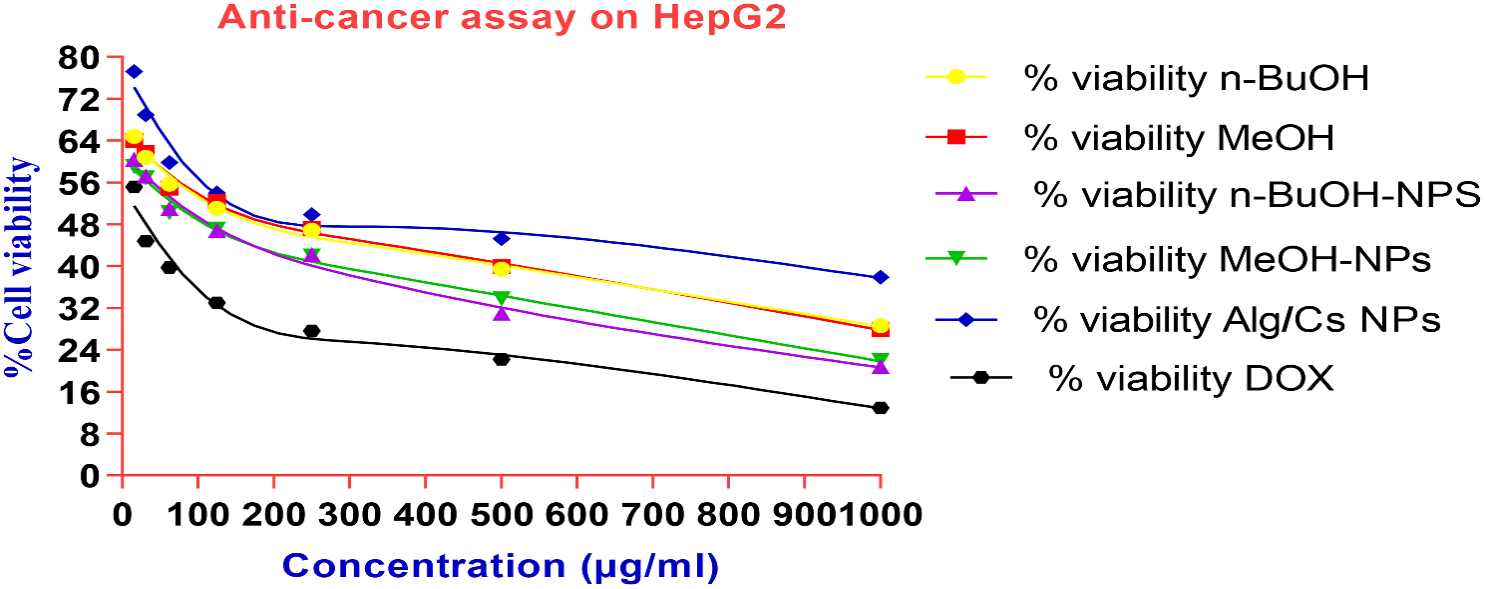
Viability % of HepG2 treated with free Alg/Cs-NPs, *n*-BuOH-NPs and MeOH-NPs and standard (Doxorubicin)

The anti-cancer activity of *C. arvensis*, *C. arvensis*-alginate/chitosan-NPs, alginate/chitosan-NPs and standard (Doxorubicin) was evaluated in vitro against HepG_2_ hepatocellular carcinoma cell lines after 24 h exposure and their IC_50_ values were determined from a graph of cell viability measured over a range of concentrations between 15.62 and 1000 µg/ml. Using the data, a line chart was created with concentrations on the X-axis and % cell viability on the Y-axis, utilizing GraphPad Prism 8. A reference line was drawn at the 50% inhibition mark on the Y-axis, which then indicated the corresponding concentration on the X-axis. (Fig. 11). From these data, it is revealed that *C. arvensis*, *C. arvensis*-alginate/chitosan- NPs, alginate/chitosan-NPs exhibited a different range of significant anti-cancer activities varying from 15.62 µg/ml to 1000 µg/ml compared to the common anti-cancer drug, Doxorubicin. It was also noticed that the encapsulation of *C. arvensis* extracts onto alginate/chitosan-NPs increased the anti-cancer activity of *C. arvensis* extracts against HepG2 hepatocellular carcinoma cell lines (Table 5; Fig. 11).

**Table 5:** The Anticancer effect of *C. arvensis*, *C. arvensis*-alginate/chitosan-NPs, alginate/chitosan-NPs on the HepG2

Concentration (µg/ml)	% cell viability					
	*n*-BuOH	MeOH	*n*-BuOH- NPs	MeOH- NPs	Alg/Cs Np	DOX
1000	28.59 ± 0.52	27.88 ± 0.68	20.80 ± 0.27	21.92 ± 0.6	37.93 ± 0.46	12.95 ± 0.42
500	39.46 ± 0.84	39.99 ± 0.75	31. 3 ± 0.45	33.73 ± 0.55	45.27 ± 0.47	22.19 ± 0.67
250	46.88 ± 1.18	47.26 ± 1.08	42.18 ± 0.61	41.98 ± 0.69	49.88 ± 0.66	27.59 ± 0.61
125	51.10 ± 1.05	52.40 ± 1.23	46.80 ± 0.87	47.10 ± 0.9	54.02 ± 0.87	32.99 ± 1.02
62.5	55.69 ± 1.14	54.92 ± 1.41	51.01 ± 1.11	50.21 ± 0.96	59.84 ± 0.69	39.76 ± 0.64
31.25	60.81 ± 1.86	61.75 ± 1.42	57.23 ± 0.93	56.93 ± 1.13	68.96 ± 0.96	44.89 ± 0.81
15.56	64.76 ± 1. 4	64.06 ± 1.38	60.45 ± 1.01	59.05 ± 1.11	77.18 ± 0.84	55.13 ± 1.04
IC_50_ (µg/ml)	141.1	153.7	93.11	86.54	168.19	22.28

### Anti-bacterial effects of *C. arvensis* extracts

**Fig. 12:**
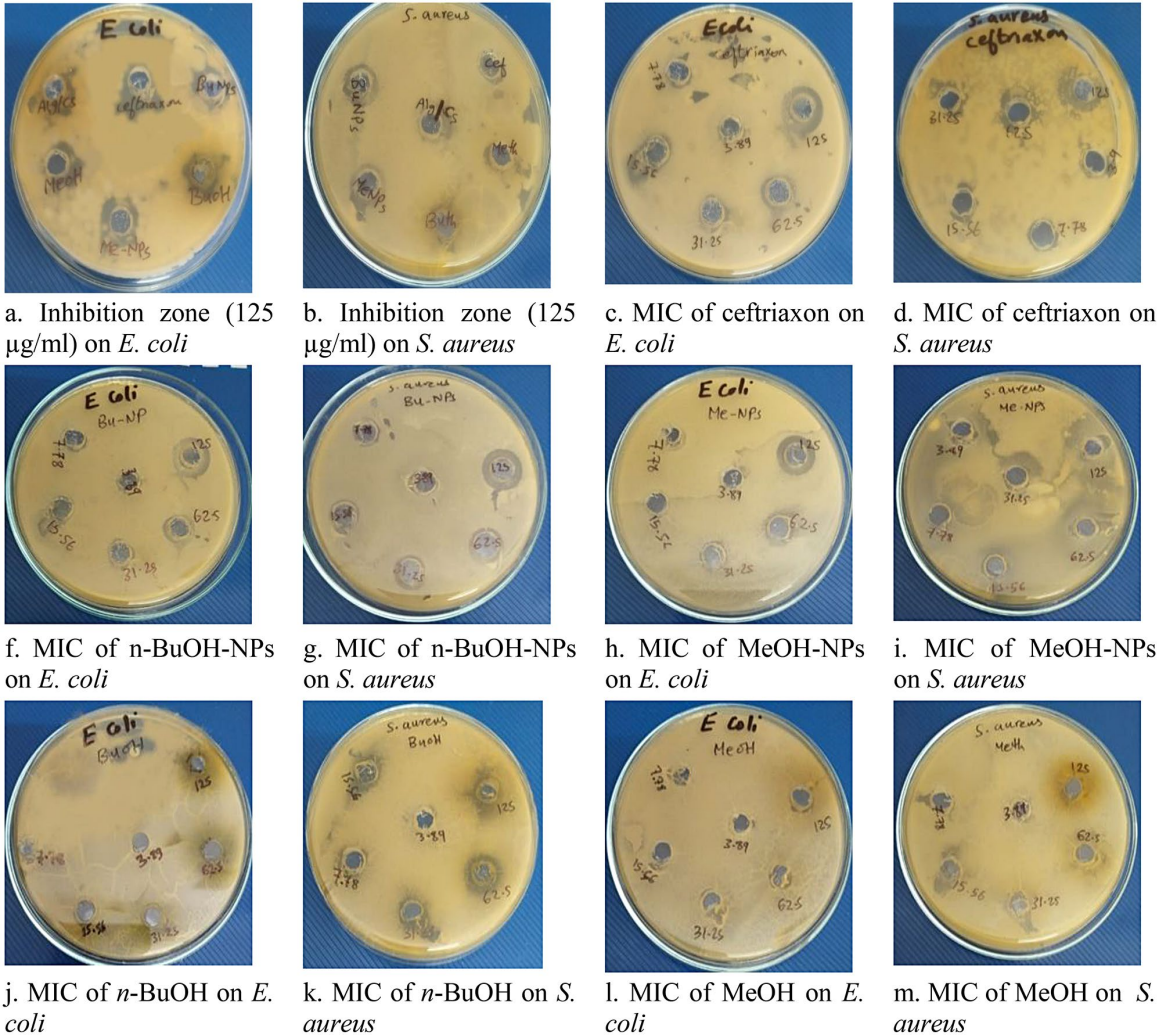
Inhibition zones of MeOH, *n*-BuOH, MeOH-NPs, *n*-BuOH-NPs and Alg/Cs-NPs compared to standard antibiotic, Ceftriaxone

The results attained in the present study indicate that the tested *C. arvensis* extracts/ NPs possess potential anti-bacterial activity against *E. coli* and *S. aureus* as shown in Table 6. The encapsulated *C. arvensis* extracts enhanced the antibacterial activity against the gram + ve and gram -ve bacteria. The results also showed that the encapsulation of *C. arvensis* extracts decreased the MIC of both extracts from 31.25 µg/ml to7.78 µg/ml in gram -ve and from 15.56 µg/ml to7.78 µg/ml in gram + ve as shown in Tables 6 and 7; Fig. 12.

**Table 6:** The Anti-bacterial effect of free Alg/Cs-NPs, n-BuOH-NPs and MeOH-NPson both *E. coli* and S. *aureus*

Bacterial strain	Inhibition zone / Concentration (125 µg/ml)					
	*n*-BuOH	MeOH	*n*-BuOH-NPs	MeOH- NPs	Alg/Cs Np	Ceftriaxone
*E. coli*	22	21	29	29	18	39
*S. aureus*	22	23	31	31	19	34

**Table 7:** The MIC of free Alg/Cs-NPs, *n*-BuOH-NPs and MeOH-NPson *E. coli* and *S. aureus*

Concentration (µg/ml)	Inhibition zone/ E. coli					
	*n*-BuOH	MeOH	*n*-BuOH- NPs	MeOH- NPs	Alg/Cs Np	Ceftriaxone
125	22	21	29	29	18	39
62.5	15	14	21	22	14	29
31.25	10	9	16	15	11	21
15.56	--	--	12	11	9	16
7.78	--	--	8	8	--	11
3.89	--	--	--	--	--	8
**Concentration** **(µg/ml)**	**Inhibition zone/** ***S. aureus***					
	***n*** **-BuOH**	**MeOH**	***n*** **-BuOH- NPs**	**MeOH- NPs**	**Alg/Cs Np**	**Ceftriaxone**
125	22	23	31	31	19	34
62.5	19	19	26	27	16	28
31.25	13	14	20	19	12	22
15.56	7	7	14	13	8	14
7.78	--	--	9	8	--	10
3.89	--	--	--	--	--	8

## Discussion

Herbal remedies are humanity’s earliest medical practices. Early civilizations heavily relied on them, and they remain the predominant form of treatment globally. Plants exhibit various health benefits such as combating microbes, reducing oxidation, fighting cancer, managing cholesterol, supporting heart and nerve functions, enhancing the immune system, reducing inflammation, and alleviating pain and fever, among others. These plants generate secondary compounds evolved from primary ones, serving as a vital foundation for numerous medicinal drugs [30].

Earlier research on *C. arvensis* identified its content of elements like alkaloids, phenolics, flavonoids, carbohydrates, sugars, mucilage, sterols, resin, tannins, unsaturated sterols/triterpenes, lactones, and proteins. Further studies indicated that *C. arvensis* has properties such as antioxidant, vasorelaxant, immune-boosting, antibacterial, anti-diarrheal, diuretic effects, and shows cytotoxicity against both human cancer cells (HELA) and Lymphoblastic Leukemia Jurkat cells [19, 24, 31, 32].

Herein, the main detected phytoconstituents using LC-ESI-MS in negative ion mode can be classified into phenolic acids, flavonoids, lipids, and terpenes (Table 1).

### Phenolic acids

The first compound of phenolic acid (t_R_= 12.02 min) was putatively assigned as quinic acid, which afforded pseudo-molecular ion at *m/z* 191 [M-H]^−^, other fragments at *m/z* 173 and 97 [25]. Compound **2** (t_R_=16.16 min) afforded peaks at *m/z* 475 [M-H]^−^, 353 [M-H-122 (benzoyl)]^−^, 191 and 137 corresponding to benzoyl-caffeoylquinic acid. Compounds **3** and **4** (t_R_=18.83 and 19.10 min, respectively) were characterized as 5-*O*-caffeoylquinic acid and 4-*O*-caffeoylquinic acid, respectively because the identical [M-H]^− ^ion is present at *m/z* 353 and same fragmentation pattern (*m/z* 191, 179, 135) but with different ion intensities [33]. Compounds 5, 7, 8 and 9 (t_R_= 20.70, 23.50, 25.23 and 26.04 min, respectively) were identified as a 3-*O*-caffeoylquinic acid dimer, 5-*O*-caffeoylquinic acid dimer, 5-*O*-caffeoylquinic acid dimer isomer and 4-*O*-caffeoylquinic acid dimer, respectively. They exhibited signal at *m/z* 707 [2 M-H]^−^ combined with additional fragments at *m/z* 353, 191, 179, and 135 resulting in the typical fragmentation pattern of caffeoylquinic acid [34, 35]. Compound 6 (t_R_= 21.23 min) afforded molecular ion at *m/z* 705 [M-H]^−^ and *m/z* 513 [M-H-192]^−^ due to neutral loss of the quinic acid unit. Further signal at *m/z* 339 [M-H-192-174]^−^ due to further loss of a dehydroquinic acid unit. Therefore, it was assigned as a 5-*O*-caffeoylquinic acid dehydrodimer [36].

Compound **11** (t_R_= 27.10 min) was pinpointed as caffeic acid based on its specific [M-H]- at *m/z* 179 and its fragmentation characteristics. While, Compound (t_R_= 2550.47 min) appeared to be a derivative of caffeic acid, given its [M-H]- at *m/z* 423. Compound 12, (t_R_= 28.30 min) was recognized as coumaroylquinic acid, evidenced by its [M-H]^−^ ion at *m/z* 337 and fragments at *m/z* 191, 173, 93, and 85. Compound 14 (t_R_= 29.77 min) afforded a molecular ion signal at *m/z* 367 [M-H]^−^ and other fragments at *m/z* 191 and 173, pinpointing as feruloylquinic acid. Lastly, Compound 22 (t_R_= 40.86 min) displayed a precursor ion at *m/z* 193 [M-H]^−^, which is typical for ferulic acid [25, 36, 37]. It was noted that caffeoylquinic acids are the most abudant phenolic acids group in *C. arvensis* 85% MeOH extract. These compounds can be classed into four major classes: mono, di, tri, and tetracaffeoylquinic acids. Also, It widely found in a variety of plants as well as in different food staff, including fruits, vegetables, coffee, and spices [38]. Further, It have a wide range of bioactivities, such as antioxidants, antibacterial, anticancer, antiparasitic, antiviral, antidiabetic, antiinflammatory, and neuroprotective effects as well as the reduction of some chronic and cardiovascular diseases [39, 40].

### Flavonoids

Most flavonoids detected in *C. arvensis* were flavonoid glycosides tentatively assigned as quercetin and kaempferol derivatives such as, compound **15** (t_R_= 33.25 min) displayed the [M-H]^−^ at *m/z* 741, 609 [M-pentosyl-H]^−^, 301[M-pentosyl-2hexosyl units -H]^−^, 179 of quercetin aglycon. Thus, this compound characterized as quercetin-*O*-pentosyl-dihexosides. Compound **17** (t_R_= 36.58 min) represented a precursor ion at *m/z* 609 [M-H]^−^ and produced fragments at *m/z* 463 and 301 revealing the loss of deoxyhexosyl and hexoside moieties, respectively. Therefore, it was assigned as quercetin-3-*O*-rutionside (rutin) which was detected in *C. arvensis* before [41]. Compound **18** (t_R_= 37.39 min) was identified as quercetin-*O*-hexoside due to presence of [M-H]^−^ at *m/z* 463 and other peaks at *m/z* 301, 179 and 151. Compound **20** (t_R_= 39.65 min) revealed a molecular ion at *m/z* 607 [M-H]^−^, that gave fragment ions at *m/z* 505 [M-H-18-44-40]^−^ revealed the loss of H_2_O, CO_2_, and C_3_H_4_, respectively. Followed by fragment ions at *m/z* 463 [M-H-18-44-40-144]^−^due to the release of 3-hydroxy-3-methylglutaryl moiety and *m/z* 301[M-H-18-44-40-144-162]^−^ which reflect a further neutral loss of hexoside unit. Hence, it assigned as quercetin-7-*O*-[3-hydroxy-3-methylglutaroyl] hexoside. In addition, all quercetin derivatives were detected in *C. arvensis* and *C. althaeoides* leaf extracts before [24, 25, 42]. Compound **16** (t_R_= 34.18 min) was tentatively identified as apigenin-C-hexoside-*O*-pentoside due to the presence of [M-H]^−^ at *m/z* 563 with distinctive fragments at 443, 413 and 293 [43]. Compound **21** (t_R_= 40.19 min) displayed the deprotonated molecular ion at *m/z* 593, and yielded a main fragment at *m/z* 285 due to loss of rutinoside moiety. Thus, this compound was assigned as kaempferol-3-*O*-rutinoside and detected in *C. arvensis* whole partsand C. *dorycnium* L. flowers extracts before [25, 44]. From the perivious data, it was observed that quercetin derivatives are the major flavonoid in this plant. Among them, quercetin-3-*O*-rutinoside (rutin) which the main constituent of *C. arvensis* as shown in Table 1; Fig. 1. Rutin is a low molecular weight flavonol-type flavonoid. It has various pharmacological properties such as antioxidant, antitumor, antiinflammatory, antibacterial, antiallergic, antiprotozoal, hypolipidaemic, cytoprotective, antispasmodic, antiviral, antiulcerogenic, and antihypertensive. Therefore, it can be found in a huge number of herbal multivitamin preparations and called as vitamin P [45].

### Other compounds

Compound **10** (t_R_= 26.57 min) afforded [M-H]^−^ at *m/z* 387 with a main fragment at *m/z* 161, which is characteristic of magastigmane glycoside derivative as reported by Abdul Khaliq et al. [25]. Megastigmanes compounds are oxygenated isonorterpenoids and frequently referred to as oxidative by-products from β-carotenoids. In addition, magastigmane glycosides have several bioactivities including antioxidant, antibacterial, antiinflammatory, anticancer, and hepatoprotective activities [46].

Compound **26** (t_R_= 58.61 min) gave [M-H]^−^ at *m/z* 327and produced major fragments at *m/z* 291, 229, 211, 171 which lead to the tentative characterization of this compound as trihydroxy-10,15- octadecadienoic acid derivative which relative to glycolipids. For our knowledge, compounds 10 and 26 were detected before in *C. arvensis* extracts [25].

Likewise, the total area percent of identified components in the 85% MeOH ext. of *C. arvensis* was 99.99% as shown in Table 1. It was noted that the phenolic acids are the highest area percent, followed by flavonoids, glycolipids, unknown compounds and sesquiterpenes, respectively as shown in Fig. 2. Among them, the major constituent in the 85% MeOH extract was rutin (33.64%) followed by 5-*O*-caffeoylquinic acid dimer (29.42%), ferulic acid (7.74%), 3, 4-di-*O*-caffeoylquinic acid (6.91%), and 4, 5-di-*O*-caffeoylquinic acid (5.81%). Hence, the bioactivities of *C. arvensis* 85% MeOH extract and its fractions may be due to their richness in polyphenolic compounds.

The cytotoxic, anti-cancer, anti-inflammatory and anti-bacterial studies were conducted on both the *C. arvensis* MeOH ext. and *n*-BuOH fr. and their loaded Alg-Cs/NPs.

At first, alginate and chitosan are examples of natural polymers that are suitable for the assembly of NPs because of their many benefits such as fast gelation, enhanced biocompatibility, and reduced toxicity. Nevertheless, they are used in hydrogel for objectives including regulating medicine release and tissue engineering [47].

The small particle size *C. arvensis* extracts loaded with Alg-Cs/NPs were expected to be lower than 100 nm in this study as a result of increasing the stirring speed of Alg-*C. arvensis* mixture before and during the addition of CaCl_2_ and chitosan solutions. In the present study, the average diameters of the prepared MeOH-NPs and *n*-BuOH-NPs were 90 ± 4.8 nm and 92 ± 5.5 nm respectively. While that of unloaded Alg/Cs polyelectrolyte NPs was 55 ± 3.9 nm, the bigger size of C. arvensis-NPs compared to empty ones indicates the incorporation of the drug inside the NPs.

Katuwavila et al. [48] designed a natural, biodegradable, and biocompatible polysaccharide nano-delivery method for the medication doxorubicin, particularly chitosan and alginate. The sizes of Cs-Alg NPs and doxorubicin loaded Cs-Alg NPs were 100 ± 0.35 and 100 ± 28.0 nm. First, chitosan droplets were formed by mixing with Tween 80. Afterward, these droplets were solidified through ionic crosslinking using an alginate solution. This step may be the reason to the smaller particle size. The larger particle size obtained by DLS measurements compared to TEM could be due to the swell ability of polymeric hydrogels in the solution as it was previously mentioned by Bhattarai et al. [49]. In a study conducted by Yin et al. [50], the team sought to create composite nanoparticles smaller than 200 nm. They examined how different component contents and process parameters impacted the particle size, swelling behavior, and Cs release rate from the carrier materials. The specific mixture chosen for their research, at a chitosan to sodium alginate ratio of 2:1, resulted in a shimmering suspension with a positive zeta potential. This combination was found optimal for encapsulating doxorubicin, achieving an impressive 95% encapsulation efficiency. Their findings align with a prior study by Sohail and Abbas, [51], where they developed a drug delivery system for amygdalin using anionic Cs-Alg NPs. Their particle sizes ranged between 119 ± 19 nm and 261 ± 18 nm, primarily targeting anti-cancer applications.

The TGA results of this study may be due to the easy loss of anion and ammonium groups in the Alg/Cs polyelectrolyte complex (Hofmann elimination) [52]. However, the nanoparticles containing *C. arvensis* extracts show that the nanoparticles possessed improved thermal stability contrasting Polymers with its polymeric nanoparticles without *C. arvensis*. This phenomenon further illustrates that a portion of *C. arvensis* accumulated on the polymer shell of the nanoparticles, which prevents the polymer shell during thermal decomposition. The results of this report agreed with the previous publication [53], who assumed that the Prussian blue formed in the polymer shell hindered the thermal degradation of polymeric nanocapsules.

The previous studies on *C. arvensis* did not evaluate the hemolytic effect on the RBCs, whereas the anti-oxidant, anti-microbial and anti-cancer activities were discussed [19, 54–56]. The CS and Alg polymers were attributed to having low toxicity and hemocompatibility [57]. San et al. [58], suggested that both the unbound TO and its combination with TO-FA-Cs/Alg-NPs demonstrated minimal hemolytic activity (5%). However, when exposed to the maximum TO concentration (7.5–10 mg/ml), this behavior changed, suggesting that they might be suitable for human use. Further tests are recommended, especially for applications involving blood contact. In this study, both the MeOH ext. and *n*-BuOH fr. of *C. arvensis* and their loaded Alg-Cs/NPs exhibited non-hemolytic activities (percent hemolysis, 3–12%) even at maximum concentration of 1000 µg/ml. The work’s outcomes concurred with those of the earlier investigation reported by Wiya et al. [59]. They stated that the hemolytic effect of the both the aqueous and ethanolic slime extracts ranged from 7.01 ± 0.54 to 13.42 ± 0.28%.

The previous studies conducted to estimate the anti-inflammatory effect of *C. arvensis*, it was observed that in aseptic arthritis brought on by carrageenan, the gel containing the extract of *C. arvensis* has an expressed anti-oxidative action. Furthermore, when administered topically to the site of pain, the anti-oxidative activity of the product containing the extract of *C. arvensis* is comparable to that of ibuprofen [56]. Another study revealed that that EtOH ext. of *C. pluricaulis Choisy* possesses strong analgesic and anti-inflammatory properties [60]. In this study, both the MeOH ext. and *n*-BuOH fr. of *C. arvensis* and their loaded Alg-Cs/NPs showed excellent anti-inflammatory activity compared to the standard drug (diclofenac).

The previous studies conducted to estimate the cytotoxic activity of *C. arvensis* showed that, the chloroform extract of *C. arvensis* exhibited a cytotoxic impact akin to taxol when tested on Hela cells (15 vs. 12 µg/ml). This heightened cytotoxicity from the chloroform extract might be due to the extraction of lipophilic glycosides by chloroform, which are non-polar compounds [31]. Moreover, anti-bacterial, anti-cancer and toxic activities of *C. arvensis*, *C. austro-aegyptiacus* and *C. pilosellifolius* extracts were tested against clinical samples, various cell types, and specific lab animals. Notably, *C. austro-aegyptiacus* displayed significant antimicrobial action. In comparison to vinblastine sulphate’s anti-tumor activity (30.3 ± 1.4) against CACO (colorectal carcinoma), both C. arvensis and *C. pilosellifolius* presented anti-tumor effects of (6.1 ± 03) and (16.4 ± 0.3), respectively [52]. Additionally, C. *arvensis’s* EtOH extract had a strong cytotoxic effect on the cancer cell line Jurkat lymphoblastic leukaemia cells. Moreover, at lower extract doses, apoptotic effects were observed, while necrotic outcomes were prominent at higher concentrations [32].

In this study, it was observed that the MeOH extract and *n*-BuOH fraction of *C. arvensis* and their loaded Alg-Cs/NPs have admirable anti-cancer activity compared to the standard drug doxorubicin. Also, the encapsulation of both the MeOH ext. and *n*-BuOH fr. of *C. arvensis* has enhanced their anti- cancer activity and lowered the IC_50_ from 153.7and 141.1 µg/ml for methanolic and *n*-BuOH extracts to 93.11and 86.54 µg/ml, alternatively. Moreover, the encapsulation of the MeOH ext. and *n*-BuOH fraction thought Alg-Cs/NPs has enhanced the safety of *C. arvensis* on normal cells and actually increased the viability of Vero cell lines by 10%.

The findings align with prior research demonstrating the anti-microbial properties of *C. arvensis* against both gram-positive and gram-negative bacteria [19, 55, 61]. The assessment of anti-microbial efficacy was performed on clinically relevant pathogenic bacteria (*E. coli and S. aureus*) by using MICs tests and disc diffusion. Nearly the same inhibition zone (22 ± 0.82 mm) against *S. aureus* compared to 21.35 ± 0.76 mm. Furthermore, the encapsulation of the aqueous and ethanolic extracts thought Alg-Cs/NPs enhanced the anti-microbial effects of *C. arvensis* and actually decreased the MIC from 31.25 to 7.78 bacteria.

The treatment’s effectiveness and cellular absorption can be significantly impacted by the size, shape, and surface chemistry of NPs revealed that the obtained C. arvensis- Alg/Cs-NPs had a suitable size for use in therapeutic applications and could be useful for the treatment of liver cancers [62].

## Conclusion

The current research revealed that the MeOH extract of *C. arvensis* is rich by bioactive phytoconstituents, majorly phenolic acids, flavonoids, glycolipid and sesquiterpene which were putatively characterized by LC-ESI-MS analysis. Both MeOH ext. and *n*-BuOH fr. were encapsulated using alginate and chitosan nanoparticles. The results revealed that *C. arvensis*-Alg-Cs/NPs extracts had more promising potential in their anti-cancer, anti-microbial and anti-inflammatory activities than the free extracts. For example, the anti-inflammatory effect of encapsulated MeOH extract (IC_50_ = 175 µg/ml) was much higher than that of free extract (IC_50_ = 1050 µg/ml). In addition, the anticancer activity of MeOH extract on HepG2 cells of encapsulated MeOH extract (IC_50_ = 86.54 µg/ml) was much high than free extract (IC_50_ = 153.70 µg/ml). Furthermore, the antibacterial activities were increased through the encapsulation process. Therefore, alginate and chitosan are excellent natural polymers that enhance the pharmacological properties of *C. arvensis* extracts and are considered a good matrix for the encapsulation of natural extracts that could be used in pharmaceutical industries.

## Data Availability

The datasets generated or analyzed during the current study are available from the corresponding author upon reasonable request.

## Abbreviations

Alg/Cs Nps: alginate/chitosan-Nano particles
*C. arvensis*: *Convolulus arvensis*
g: gram
LC-ESI-MS: Liquid chromatography electrospray ionization mass spectrometry
MeOH: Methanol
mg: Milligram
*n*-BuOH: Normal butanol
pet-ether: Petroleum ether
t_R_: Retention time
TIC: Total ion chromatogram
PBS: Phosphate buffer saline
FBS: Fetal bovine serum
RBCs: Red blood cells
